# Who Follows eHealth Interventions as Recommended? A Study of Participants' Personal Characteristics From the Experimental Arm of a Randomized Controlled Trial

**DOI:** 10.2196/jmir.3932

**Published:** 2015-05-11

**Authors:** Dominique A Reinwand, Daniela N Schulz, Rik Crutzen, Stef PJ Kremers, Hein de Vries

**Affiliations:** ^1^CAPHRI School for Public Health and Primary CareDepartment of Health PromotionMaastricht UniversityMaastrichtNetherlands; ^2^Jacobs Center for Lifelong Learning and Institutional DevelopmentJacobs UniversityBremenGermany; ^3^NUTRIM School for Nutrition, Toxicology and MetabolismDepartment of Health PromotionMaastricht UniversityMaastrichtNetherlands

**Keywords:** eHealth, Web-based intervention, intervention use, computer tailoring, personal characteristics, health lifestyle, multiple health behaviors, intervention adherence, socioeconomic status

## Abstract

**Background:**

Computer-tailored eHealth interventions to improve health behavior have been demonstrated to be effective and cost-effective if they are used as recommended. However, different subgroups may use the Internet differently, which might also affect intervention use and effectiveness. To date, there is little research available depicting whether adherence to intervention recommendations differs according to personal characteristics.

**Objective:**

The aim was to assess which personal characteristics are associated with using an eHealth intervention as recommended.

**Methods:**

A randomized controlled trial was conducted among a sample of the adult Dutch population (N=1638) testing an intervention aimed at improving 5 healthy lifestyle behaviors: increasing fruit and vegetable consumption, increasing physical activity, reducing alcohol intake, and promoting smoking cessation. Participants were asked to participate in those specific online modules for which they did not meet the national guideline(s) for the respective behavior(s). Participants who started with fewer than the recommended number of modules of the intervention were defined as users who did not follow the intervention recommendation.

**Results:**

The fewer modules recommended to participants, the better participants adhered to the intervention modules. Following the intervention recommendation increased when participants were older (χ^2^_1_=39.8, *P*<.001), female (χ^2^_1_=15.8, *P*<.001), unemployed (χ^2^_1_=7.9, *P*=.003), ill (χ^2^_1_=4.5, *P*=.02), or in a relationship (χ^2^_1_=7.8, *P*=.003). No significant relevant differences were found between groups with different levels of education, incomes, or quality of life.

**Conclusion:**

Our findings indicate that eHealth interventions were used differently by subgroups. The more frequent as-recommended intervention use by unemployed, older, and ill participants may be an indication that these eHealth interventions are attractive to people with a greater need for health care information. Further research is necessary to make intervention use more attractive for people with unhealthy lifestyle patterns.

## Introduction

New eHealth interventions are an important tool to improve public health by providing people with information, skills, and support needed for a positive health-related lifestyle change [1,2]. These eHealth interventions provide the opportunity to use computer tailoring to provide highly personalized information to a respondent without face-to-face counseling [3-5]. With the use of computer tailoring, participants receive information derived from an individual assessment, attuned to their individual answers, and, in our case, aimed at motivating individuals to adopt 1 or more healthy behaviors [6]. Consequently, computer-tailored interventions provide feedback that is more relevant to the individual, contains less redundant information, and is more likely to be processed and remembered than generic information [7-10]. Furthermore, eHealth interventions are easily accessible and have the potential to reach a wide population [11,12].

Numerous studies have demonstrated that computer-tailored interventions are effective in motivating individuals to adopt health behaviors [1,13-15], including increased physical activity [16-20], healthy nutrition [5,20-24], smoking prevention and cessation [25-28], and decreasing alcohol intake [29-31]. Moreover, interventions to change multiple health behaviors have also been shown to be effective [32-35]. Studies also showed that computer-tailored interventions are more cost-effective than typical health care [36-38].

People of a low socioeconomic status (SES) often have unhealthy lifestyle behaviors [39,40]. They often eat fewer fruits and vegetables [41], are less physically active (eg, [42]), consume more alcohol (eg, [43]), and smoke more tobacco (eg, [44]) compared to people with higher SES.

Although there is evidence that Web-based interventions are effective in improving health behavior, these interventions come with high dropout rates and the problem that participants often do not use the intervention as recommended [45-47]. Although studies have investigated characteristics of dropout and nonusage of eHealth interventions (eg, [45,48]), it is equally important to know more about the participants who use these interventions as recommended. It is conceivable that people with different sociodemographic profiles may use these Web-based interventions differently because of general differences in online behavior and Internet usage between certain groups. This digital divide, for example, refers to unequal access to and use of the Internet among people with a lower SES [49-53].

More than 90% of the general Dutch population has access to the Internet and 86% of Dutch people use the Internet every day [54,55]. Although the gap between people with and without Internet access seems to be closing, there is still a difference in Internet use between certain sociodemographic groups [56]. People with a higher SES use the Internet more often than people with a lower SES to achieve personal development (eg, getting a new job), whereas people with a lower SES use the Internet primarily for other purposes, such as entertainment [57,58].

Education is often used as a proxy to measure SES; therefore, the literature about education and Internet use is extensive. People with a higher educational level have been found to use the Internet more frequently to gain health-related information, for work, and for shopping or product information. People with a lower educational level use the Internet more often with other objectives, such as browsing the Web or playing online games [59-62]. Educational level might also play a role in online behavior because most information on the Internet is written at a high literacy level whereas nearly half of the people do not understand this level of written information [63]. Moreover, employed people spend less of their leisure time online [57]. People with a lower income use the Internet more often for entertainment purposes, such as downloading music [64-66], whereas people with a higher income spend more of their time online searching for news or information [57].

Age- and gender-based differences in Internet use also exist. Although the majority (80%) of Dutch people aged 65 years or older have access to the Internet [67], they are less familiar with routine daily use of the Internet [56,68] and use the Internet primarily as an information source [69] in contrast to younger Internet users who primarily understand the Internet as an entertainment medium [70]. Males have been found to use the Internet more often, are more experienced with Internet use, and feel more comfortable with it [56,71]. This is in contrast to women, who spend less time online when having to take care of their family [72], but seek health information online more often than men [64]. In addition to these, other personal factors, such as a lower quality of life [73,74] or being married, are related to less Internet usage [75].

Because SES is an important predictor of how people use the Internet [56], it is conceivable that people with a lower SES may not implement eHealth interventions as intended by the intervention developer and may be unlikely to follow intervention recommendations, which makes behavior change less likely [76]. However, because people with a lower SES are a high-risk group for unhealthy behaviors [44,77-79], they are a highly relevant target group that might benefit from eHealth behavior change interventions. The same reasoning might hold for other personal characteristics, such as age, perceived health, or quality of life.

Therefore, this study investigates whether people are following the recommendations of how to use eHealth interventions. The purpose of our study is to identify personal and socioeconomic characteristics associated with recommended eHealth intervention use. Based on findings from the literature, we hypothesize that people with a higher education, who do not have paid work, those who have a lower income, who are younger, female, have a high perceived quality of life, and are not in a relationship are more likely to use the intervention as recommended.

## Methods

### Overview

A detailed description of the study protocol has been published elsewhere [80]; only those study methodology details relevant to the study at hand are described here.

### Participants, Procedure, Study Design, and Intervention Content

This study is part of a randomized controlled trial that was conducted in the Netherlands between 2009 and 2012. The study received ethics approval from the Medical Ethics Committee of Maastricht University and the University Hospital Maastricht (MEC) and has been registered by the Dutch Trial Register (NTR 2168). Participants were recruited through different Dutch Regional Health Authorities (RHAs) in the Netherlands [80]. These RHAs periodically monitor the health status, health behaviors, and related aspects of the adult population. At the end of this monitor*,* people were asked if they were interested in participating in this study. They were told that they would be invited to take part in a free online program that provides participants with tailored feedback about their health behavior. Internet access, a computer, basic Internet skills, and sufficient Dutch language skills were required preconditions for participating. The intervention consisted of 2 parts and focused on 5 health behaviors: fruit consumption, vegetable consumption, physical activity, smoking behavior, and alcohol intake. During the first part of the intervention, participants had to answer questionnaires about their health behaviors. The answers were used to provide participants with their personalized risk appraisal, which provided feedback by comparing the respondents’ behavior to the Dutch guidelines defined for the 5 behaviors, such as (1) being physically active for at least 30 minutes on at least 5 days a week, (2) eating at least 200 grams of vegetables, (3) eating at least 2 pieces of fruit each day, (4) drinking no more than 2 glasses of alcohol a day (for men; 1 glass for women), and (5) not smoking at all.

The second part of the intervention consisted of 5 lifestyle modules. Participants who were interested in participation in the program received an email with their personalized link to log on to the computer-tailoring program. Based on the first part, the questionnaire assessment as part of the RHA monitor, participants were provided with tailored feedback concerning their behavior. They received an overview about all 5 behaviors and whether they met the guidelines or not. In the second part of the program, participants were asked to complete all modules for which they did not meet the guidelines. For example, in case a participant reported smoking and eating less than 2 pieces of fruit a day, he/she was advised to participate in the modules for smoking and fruit consumption. All modules included tailored feedback based on the determinants specified in the I-Change Model [81]: attitudes, social influence, self-efficacy, and preparatory and coping planning. The order of the modules was counterbalanced, either starting with preventive behaviors and addiction behaviors (ie, physical activity/vegetable consumption/fruit consumption and alcohol intake/smoking), or vice versa (alcohol intake/smoking followed by physical activity/vegetable consumption/fruit consumption).

### Measures

#### Demographic Information

The following demographic information were assessed: age, gender (1=male; 2=female), education (1=low: no education, primary, or lower vocational school; 2=middle: secondary vocational school or high school; 3=high: higher professional education or university), monthly income (1≤€1751; 2=€1751-€3050; 3≥€3050), work situation (1=no paid job; 2=paid job) [82], family status (1=single; 2=relationship), number of persons living in the household, and country of birth (1=the Netherlands; 2=other).

#### Health Status

Participants were asked whether they suffered from (any of) the following diseases: diabetes mellitus, myocardial infarction, stroke, high blood pressure, other cardiovascular diseases, and/or cancer. Participants were categorized as ill (1=suffering from at least 1 of the diseases) or healthy (0). To assess quality of life, the Short Form Health Survey (SF-12) questionnaire was used [83-85] (ranging from 18 to 48; based on a mean split of 24, we defined 0=a low quality of life score and 1=a high quality of life score).

#### Health Behaviors

All 5 health behaviors were assessed with the use of validated questionnaires. Physical activity was assessed with the Short Questionnaire to Assess Health-Enhancing Physical Activity (SQUASH) [86]. Weekly vegetable intake (raw, boiled, baked, or salad), weekly fruit intake, and fruit juice consumption were assessed with the Food Frequency Questionnaire (FFQ) [87]. Alcohol consumption was assessed with the 5-item Dutch Quantity-Frequency-Variability (QFV) questionnaire [88]. Smoking behavior was assessed by asking if, what (eg, cigarettes, shag), and in what quantities participants smoke and their answers were converted into a score for tobacco consumption according to the recommendations by Mudde and colleagues [89].

#### Intervention Use

We defined someone as using the intervention as recommended if he/she started with the suggested number of lifestyle modules based on his/her assessed behavior. Answering the first question within the specific module was defined as starting the module. For example, if a participant did not meet the guideline for smoking, vegetable consumption, and physical activity, this person was expected to start with 3 lifestyle modules (more than 3 modules were also counted as using the intervention as recommended) to be classified as a participant who uses the intervention as recommended. If this participant only started 2 or less modules, he/she was classified as not meeting the intervention recommendation.

### Statistical Analyses

The data was analyzed with SPSS 20.0 (IBM Corp, Armonk, NY, USA). Descriptive statistics were used to describe participants’ characteristics.

Frequency analyses were performed to identify the number of participants who used the intervention as recommended (1) and those who did not (0), as defined by the intervention recommendation. Only participants who got the advice to complete at least 1 module were included in analysis of intervention use as recommended. Differentiations were made between different subgroups for age, gender, education, income, working situation, health status, family status, and quality of life. We used chi-square tests to explore the differences among these groups.

Logistic regression analyses using the Enter method were used to predict intervention use as recommended among different personal characteristics. The number of the received intervention modules, based on the amount of health behaviors that did not met the national guidelines, was used as a dependent variable. Separate logistic regression analyses were carried out dependent on the amount of lifestyle modules participants were advised to complete. One logistic regression analysis including all respondents was conducted with as-recommended program use as a dependent variable to detect characteristics that predict intervention use in general. Age, gender, level of education, income, employment status, family status, household size, country of birth, diseases, and quality of life were included as predictors in all models. The recommended number of modules was also taken into account in the model. Tests were performed at alpha=.05 to indicate statistical significance.

## Results

### Sample Characteristics

A total of 1638 participants were included in this study (Table 1). Variables with missing data were not filled up (maximum missing values of 2.4%). The mean age was 43.9 years (SD 12.6) and slightly more men (53.60%, 878/1638) than women (46.40%, 760/1638) participated in the trial. A minority of the participants had a low educational level (10.84%, 174/1605). The majority had a middle income (46.83%, 767/1606), were in a relationship (75.94%, 1215/1600), and came originally from the Netherlands (95.27%, 1531/1607). Most participants were healthy (79.84%, 1283/1607) and reported a high quality of life (58.51%, 935/1598).

### Intervention Use

**Figure 1:**
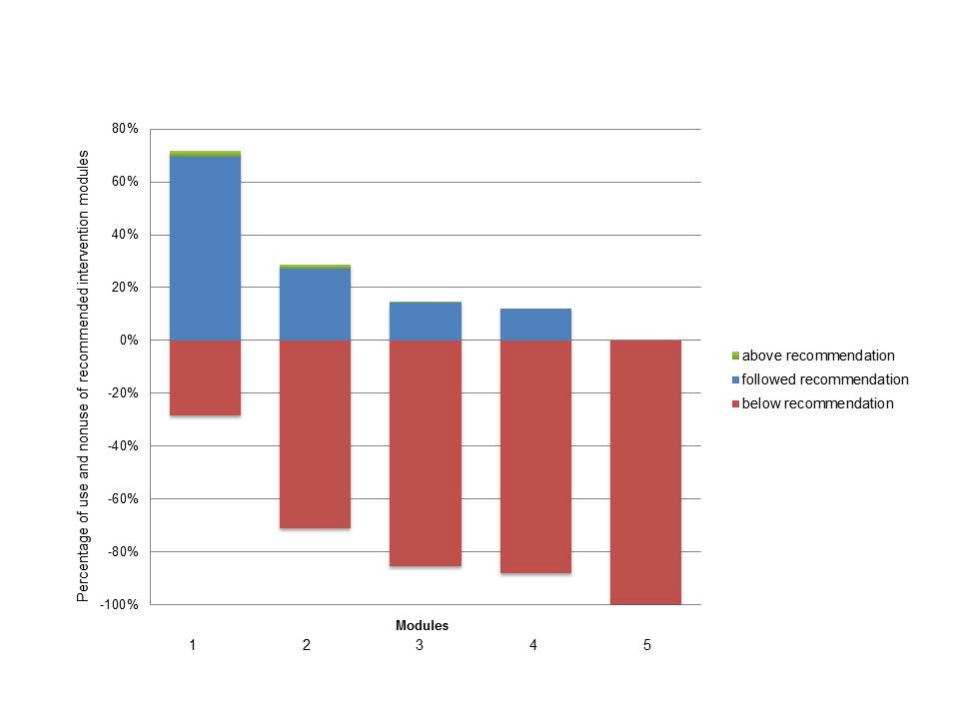
Percentage of participants who used the intervention in the recommended way.

Figure 1 represents the percentages of participants who used the intervention in the recommended way. For example, 414 of 585 participants (70.8%, red bar) who were advised to complete at least 2 lifestyle modules did not do so, 162 participants (27.7%, blue bar) followed the recommendation, and a minority (9/585, 1.5%, green bar), attended more than 2 modules. These percentages indicate that the healthier their lifestyle and the fewer modules participants were advised to complete, the more participants followed the intervention guideline.

**Table 1:** Sample characteristics (N=1638).

Characteristics		n (%)	Mean (SD)	Range
Age (years)			43.94 (12.57)	19-65
**Gender**				
	Male	878 (53.60)		
	Female	760 (46.40)		
**Educational level**				
	High	700 (43.16)		
	Middle	731 (45.55)		
	Low	177 (10.84)		
**Income (€)**				
	<1750	373 (22.77)		
	1751-3050	767 (46.83)		
	>3051	466 (28.45)		
**Working situation**				
	Paid job	1240 (77.26)		
	Nonpaid job	365 (22.74)		
**Family status**				
	Single	385 (24.06)		
	In relationship	1215 (75.94)		
Number of people in household			2.89 (1.37)	1-11
**Country of birth**				
	The Netherlands	1531 (95.27)		
	Other	76 (4.73)		
**Disease status**				
	Ill	324 (20.16)		
	Healthy	1283 (79.84)		
**Quality of life (SF-12)**			40.19 (5.08)	18-48
	High	935 (58.51)		
	Low	663 (41.49)		
**Number of modules recommended**				
	0	174 (10.62)		
	1	451 (27.53		
	2	585 (35.71)		
	3	315 (19.23)		
	4	100 (6.11)		
	5	13 (0.79)		

### Intervention Use by Different Subgroups

**Figure 2:**
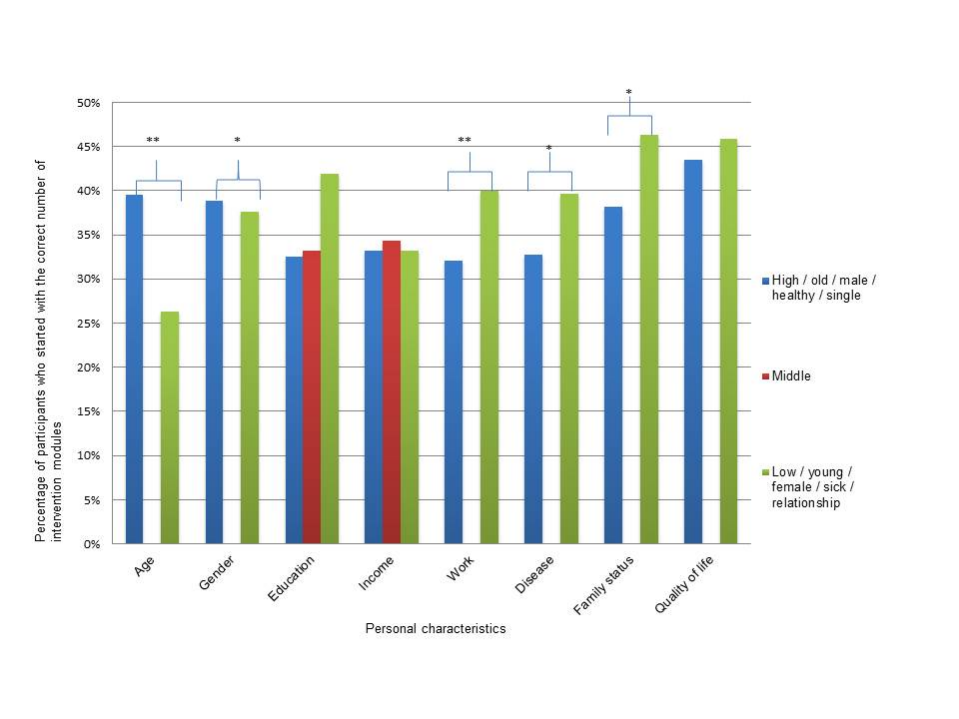
Percentage of participants who followed the recommendation to start with the correct number of intervention modules differentiated by education, income, work, age, gender, and disease status. Age was categorized as 1=young and 2=old based on a mean split of 44 years. *P<.05, **P<.001.

The table in Multimedia Appendix 1 gives an overview of the number of unhealthy behaviors and the number of started modules, differentiated by several personal variables. Figure 2 graphically summarizes the difference between the subgroups and the number of participants who did not comply with the intervention recommendation compared to those who used the intervention as recommended.

Significantly more older (39.50%, 361/914) than younger participants (26.3%, 191/726; χ^2^_1_=44.8, *P*<.001) and significantly more women (50.66%, 385/760) than men (38.8%, 341/878; χ^2^_1_=23.0, *P*<.001) used the intervention as recommended.

People with low education (42.0%, 73/174) adhered best to the recommendation to participate in the suggested modules depending on their health behavior compared with participants with middle (33.2%, 243/174) or high education (32.6%, 228/700). However, no significant differences among these 3 educational levels were found with regard to recommended intervention use (χ^2^_1_=2.9, *P*=.23).

Participants with a low income (33.24%, 124/373), middle income (34.29%, 263/767), and high income (33.26%, 155/466) also did not differ significantly from one another with regard to intervention use (χ^2^_2_=0.6, *P*=.72).

However, participants without a paid job (40.0%, 146/365) followed the recommendation of the intervention significantly more often than participants with a paid job (32.02%, 397/1240; χ^2^_1_=7.9, *P*=.01).

Those participants who were ill (37.7%, 123/324) did use the intervention as recommended significantly more often compared to healthy participants (32.74%, 420/1283; χ^2^_1_=4.5, *P*=.02).

Participants in a relationship (46.34%, 563/1215) followed the intervention recommendation significantly more often than single participants (38.2%, 147/385; χ^2^_1_=7.8, *P*=.003).

Finally, no significant differences were found for participants with a high quality of life (43.5%, 407/935) compared to those with a low quality of life (45.9%, 304/663; χ^2^_1_=0.9, *P*=.19).

### Predictors of Intervention Use

As indicated in Table 2, higher age at baseline was a significant predictor of following the relevant recommendation to start with 1 or more modules. This was found for those who were recommended to follow 1, 2, and 3 modules, and in general. Age did not predict intervention use as recommended for respondents who were recommended to follow 4 lifestyle modules.

**Table 2:** Logistic regression results for the relationship between socioeconomic variables, personal characteristics, and following the intervention recommendation.

Predictor^a^		Number of modules recommended to start with											
		1 module (n=427)			2 modules (n=556)			3 modules (n=302)			4 modules (n=108)		
		β	*P*	OR (95% CI)	β	*P*	OR (95% CI)	β	*P*	OR (95% CI)	β	*P*	OR (95% CI)
Age (cont)		0.04	<.001	1.05 (10.02-10.07)	0.04	<.001	1.04 (1.02-1.05)	0.05	.002	1.06 (1.02-1.09)	–0.03	.30	0.97 (0.90-1.04)
Gender (ref=female)		0.17	.62	1.13 (0.71-10.78)	0.54	.007	1.16 (1.24-2.54)	0.41	.22	1.60 (0.76-3.24)	–0.91	.24	0.41 (0.05-3.07)
Diseases (ref=healthy)		0.08	.82	1.09 (0.57-2.04)	–0.17	.50	0.84 (0.51-1.39)	0.40	.24	1.72 (0.69-4.2)	–1.36	.37	0.26 (0.05-1.41)
Country of birth (ref=other than NL)^b^		–0.32	.52	0.73 (0.27-1.95)	–0.46	.31	0.63 (0.27-1.53)						
Family status (ref=relationship)		–0.68	.03	0.50 (0.27-0.94)	0.35	.21	1.42 (0.82-2.25)	0.68	.16	2.12 (0.71-6.03)	–1.24	.41	0.29 (0.04-2.18)
Household (cont)		–0.10	.25	0.90 (0.76-1.07)	–0.04	.69	0.96 (0.83-1.13)	–0.12	.42	0.88 (0.66-1.21)	–0.80	.07	0.45 (0.20-1.05)
**Income (ref=low)**													
	Low		.33			.25			.59			.12	
	Middle	0.46	.21	1.59 (0.77-3.29)	–0.48	.15	0.62 (0.33-1.2)	–0.16	.53	0.67 (0.26-2.85)	–1.25	.15	0.06 (0.02-4.18)
	High	0.37	.17	1.45 (0.85-2.50)	–0.36	.13	0.70 (0.43-1.11)	0.21	.81	1.12 (0.51-2.99)	1.01	.55	1.86 (0.39-19.44)
Work situation (ref=unemployed)		–0.23	.45	0.79 (0.44-1.44)	–0.001	.99	0.99 (0.62-1.61)	–0.27	.68	0.84 (0.36-1.9)	–1.26	.14	0.20 (0.04-1.99)
**Education (ref=low)**													
	Low		.65			.74			.70			.08	
	Middle	0.15	.73	1.16 (0.50-2.70)	–0.26	.44	0.77 (0.40-1.5)	0.40	.40	1.60 (0.57-4.67)	2.84	.08	10.99 (1.38-212.83)
	High	0.24	.36	1.27 (0.77-2.08)	–0.06	.79	0.94 (0.62-1.49)	0.08	.71	1.17 (0.52-2.54)	–0.26	.65	0.65 (0.13-4.65)
QOL (cont)		–0.02	.37	0.98 (0.93-1.03)	–0.07	.002	0.93 (0.89-0.98)		.22	0.96 (0.9-1.03)	–0.09	.24	0.92 (0.8-1.06)

^a^Cont=continuous; ref=reference group for categorical variables.

^b^ Analysis of country of birth not possible for those in 3 and 4 modules because number of participants not from the Netherlands<10.

Being single significantly predicted recommended intervention use, but only for those participants who were advised to start with 1 module. However, none of the socioeconomic variables (education, income, and work) had a significant influence on the intervention use behavior regardless of the number of unhealthy behaviors.

A low quality of life (SF-12) was associated with being more likely to use the intervention as recommended for only people who received the advice to start with 2 modules. It should be noticed that the analysis of country of birth was not possible for the model with 3 and 4 recommended modules because the number of participants not from the Netherlands was less than 10.

The regression analysis of intervention use in general indicated that being older, female, having a lower quality of life, and given the recommendation to complete fewer lifestyle modules were significant predictors of intervention use according to recommendations (see Table 3).

**Table 3:** Logistic regression results for the relationship between socioeconomic variables, personal characteristics, and following the intervention recommendation within the complete sample (N=1586).

Predictor		β	*P*	OR (95% CI)
Age (cont)		0.04	<.001	1.04 (1.02-1.05)
Gender (ref=female)		0.34	.02	1.40 (1.08-1.80)
Diseases (ref=healthy)		–0.18	.71	0.94 (0.67-1.31)
Country of birth (ref=not NL)		–0.26	.40	0.77 (0.42-1.41)
Family status (ref=in relationship)		0.01	.97	1.0 (0.67-1.46)
Household (cont)		–0.09	.07	0.91 (0.82-1.01)
**Income (ref=high)**				
	Low		.67	
	Middle	–0.16	.47	0.86 (0.56-1.31)
	High	0.01	.98	1.06 (0.74-1.34)
Work situation (ref=unemployed)		–0.15	.35	0.86 (0.62-1.19)
**Education (ref=high)**				
	Low		.78	
	Middle	0.06	.79	1.06 (0.68-1.67)
	High	0.08	.58	1.08 (0.82-1.43)
QOL (cont)		–0.04	.002	0.96 (0.93-0.98)
Module recommendation (cont)		–1.59	<.001	0.20 (0.17-0.24)

^a^ Cont=continuous; ref=reference group for categorical variables.

## Discussion

### Principal Findings

Because eHealth intervention use as recommended increases the effectiveness of behavior change [76], it is of high importance that people at a high risk of unhealthy lifestyle behaviors use those interventions in such a way. Our analysis of a sample of the general Dutch population revealed that there was a difference in intervention use among people grouped by different personal characteristics.

Contrary to earlier findings regarding Internet use and age (eg, [56,59]), we found that more older than younger participants used the intervention as recommended. It might be possible that older people were less familiar with eHealth interventions and, therefore, gained more information that was new and relevant to them resulting in more frequent use of the intervention modules.

Women in our study used the intervention as recommended more often, which could be explained by the fact that women use the Internet to seek health information more frequently than men [90]. Females tend to be more interested in health topics [91,92] and rely more often on the Internet as a trustful source [59,93]. These explanations might be possible reasons why women used the intervention as recommended more frequently compared to men in our study. In addition, males have been found to evaluate the Internet as a less valuable source of health information than women do [59], which might include eHealth interventions, and this may be another reason for the lower intervention adherence by men in our study.

Participants within a relationship have been found to use the intervention as recommended more frequently compared to singles. People within a relationship have been found to have healthier behavior and health might be something in their interest which could explain why they are more interested in using the intervention [94]. Further research should explore the importance of family status as well as health behaviors of other family members in more depth because it might be that family members are more likely to behave alike, which might ultimately affect (the need for) intervention use.

People with a lower educational level used more intervention modules than those with a higher education level did. This result is surprising because it is known from the literature that higher-educated people spend more time online to seek health information [62,95]. One explanation is that the lower-educated participants may lack prior knowledge and may have used this intervention to gain more knowledge about a healthy lifestyle [59]. Another explanation could be that people with a lower SES use the Internet primarily to gain information, whereas people with a high SES make use of different sources, including professionals or their social environment, and thus rely less on the Internet for information [93]. But these results must be interpreted with caution because although we found higher as-recommended intervention use, education was not a significant predictor within the regression analysis. Furthermore, our data indicated that income is not a predictor for recommended intervention use, which might indicate that income level might not be important with regard to intervention use as recommended.

This study revealed that unemployed people used the intervention as recommended more frequently. Participation in the intervention is time-consuming and it may be that employed people adhered less to the intervention recommendations because they had less leisure time. Previously, van Deursen and van Dijk [57] reported that unemployed people spent more time online than employed people did.

The fact that participants who reported having a disease used our intervention more frequently is in-line with previous literature findings. Individuals who perceive themselves as more ill have been found to use the Internet as a source of health information [96,97]. This might also be an explanation for our finding that participants with a lower level of quality of life used the intervention as recommended. It might be plausible that these participants look for health information and tips about how to change their lifestyle to gain a better health condition and a higher quality of life.

We also found the more modules recommended to complete, the fewer were done by participants. Following the recommendations of an eHealth intervention requires a significant investment of time for reading and processing information and interacting with the program. Participants who received the recommendation to use many modules might be at greater risk of being overwhelmed by those requirements. If an eHealth program demands too much cognitive effort from their participants, ego depletion [98,99] can arise and participants might be more inclined not to use the program as recommended.

To summarize, we have found differences in intervention use as recommended among participants with different personal characteristics. We know that especially younger people, males, people who have a job, people with illnesses, and singles did not use the intervention as recommended. Furthermore, our analysis revealed that being older, female, having a low quality of life, and a healthier lifestyle are predictors of intervention use as recommended when all personal characteristics are taken into account.

### Strengths, Limitations, and Further Implications

One of the strengths of this study is the multiple-behavior approach because previous studies have demonstrated that those interventions have a high impact on behavior change [2]. Furthermore, we assessed several indications to measure SES, which allows us to compare the impacts of education, income, and occupational status.

In addition to the randomization of the started behaviors, either preventive or addictive modules, one of the limitations of this study is that participants could not choose on their own which module they wanted to begin with in the given module block. This might have increased the risk of participants not using the intervention because they might have disliked a given sequence. Furthermore, a predefined order of the modules may have led to reduced feelings of choice. We were not able to analyze potential consequences of this reduction and further studies may explore this issue at greater depth. Participants could have also misinterpreted a predefined order as an order of importance. Providing the module about fruit consumption first and then smoking might have created the impression that changing fruit consumption is more important than smoking cessation. Another limitation of the predefined order of the modules might be that some participants had to start with a behavior that they experienced as difficult, which may have led to reduced motivation to complete the full program. Future intervention studies should study under which conditions the utilization of a simultaneous strategy favoring multiple behaviors simultaneously or sequentially are preferred by participants.

Although this study used the term “following interventions as recommended,” we can only say for sure that participants started with the recommended amount of modules and not whether they finished the modules or how they used the modules. Next, the aim of the study was to look at differences in people of this intervention group in regards to following intervention recommendations. Yet, participants of an intervention are often preselected, implying that people who are healthier and more interested in health are more willing to participate.

Another limitation of the study at hand is the use of self-reported questionnaires, which could result in an overestimation of healthy lifestyles and participants being given the recommendation to start with fewer intervention modules. Self-reported questionnaires with regard to education, income, and working situation might also result in an underestimation of people with a low SES (eg, false information about income).

Finally, we did not analyze whether following the intervention recommendation resulted in behavior change or not. However, in one of our studies [2], it was investigated that a longer visiting time and greater number of visits in the intervention resulted in higher likelihood of behavior change. To our knowledge, this is one of the first studies focusing on socioeconomic and personal characteristics as a predictor of recommended intervention use. Because we found that older participants, females, unemployed people, ill participants, and people in a relationship more often use the intervention as recommended, we should ask ourselves what strategies can be taken to make interventions more attractive to use for those who did not use the intervention as recommended. Our intervention included several strategies that are known to increase intervention use, such as giving tailored feedback, using goal-setting strategies, action planning, self-efficacy monitoring, and the use of reminders [100,101]. On the other hand, the intervention might be more attractive if interactive elements and communication tools to facilitate social support were used, when involving the social environment, or if entertaining elements (eg, additional games, quizzes) were added [100,101]. Furthermore, the health modules were very similar in terms of structure and type of feedback, and it might be that participants disliked the repetition (which also increases participants’ burden). Future research is warranted to investigate whether improving the flow experiences of participants by using strategies to attract participants’ attention and make interventions more entertaining increases recommended intervention use [102,103].

### Conclusion

Our findings indicate that different subgroups use eHealth interventions in different ways. The more frequent as-recommended intervention use by unemployed, older, and ill participants may be an indication that these eHealth interventions are attractive to people with a greater need for health care information. Therefore, computer-tailored eHealth interventions might be a promising tool to increase health status and maintain healthy lifestyles.

## Multimedia Appendix 1

Number of started intervention lifestyle modules separated personal characteristics.

## Abbreviations

FFQ: Food Frequency Questionnaire
RHA: Regional Health Authorities
SES: socioeconomic status
SF-12: Short Form Health Survey questionnaire
SQUASH: Short Questionnaire to Assess Health-Enhancing Physical Activity
QFV: Quantity-Frequency-Variability
