# Supplementary material for: Who Follows eHealth Interventions as Recommended? A Study of Participants' Personal Characteristics From the Experimental Arm of a Randomized Controlled Trial
Source: J Med Internet Res. 2015 May 11;17(5):e115. doi: 10.2196/jmir.3932 (PMC4468602; doi:10.2196/jmir.3932)
Supplement: Supplementary file 1 [file jmir_v17i5e115_app1.pdf]

Table 2. Number of started intervention lifestyle modules separated personal characteristics (the left column shows the amount of recommended modules)

Number of started modules %

Age

	0		1		2		3		4		5	
	[a] Young	Old	Young	Old	Young	Old	Young	Old	Young	Old	Young	Old
1	41.0	18.8	57.4	78.5	1.0	2.7	.5	-	-	-	-	-
2	41.7	28.2	36.7	36.5	20.8	33.1	0.8	2.1	-	-	-	-
3	49.0	40.5	36.9	34.2	4.5	5.7	8.9	19.6	06	-	-	-
4	59.5	44.8	23.8	36.2	4.8	3.4	-	3.4	11.9	12.1	-	-
5	50.0	60.0	50.0	40.0	-	-	-	-	-	-	-	-

Gender

	0		1		2		3		4		5	
	Male	Female	Male	Female	Male	Female	Male	Female	Male	Female	Male	Female
1	30.0	26.9	67.3	71.4	2.3	1.7	-	-	-	-	-	-
2	40.2	27.4	34.7	38.7	23.5	32.5	1.6	1.5	-	-	-	-
3	47.5	40.2	35.9	35.0	3.5	7.7	12.6	17.1	.5	-	-	-
4	51.5	50.0	29.4	34.4	2.9	6.3	1.5	3.1	14.7	6.3	-	-
5	33.3	100	66.7	-	-	-	-	-	-	-	-	-

Education

	0			1			2			3			4			5		
	L	M	H	L	M	H	L	M	H	L	M	H	L	M	H	L	M	H
1	20.8	26,2	32	77	70.8	66.5	2.1	2.6	1	-	0.5	-	-	-	-	-	-	-
2	26.9	29.8	39.8	40.3	40.9	31	29.9	27.7	28.4	3.0	1.7	0.8	-	-	-	-	-	-
3	45	47	43.3	25	43.9	39.2	7.5	2.7	5.8	22.5	15.4	10.3	-	-	0.8	-	-	-
4	11.1	58.6	48.4	44.4	25.9	35.5	44.4	5.2	3.2	-	1.7	3,2	-	8.6	9.7	-	-	-
5	-	60	66.7	100	40	33.3	-	-	-	-	-	-	-	-	-	-	-	-

Income

	0			1			2			3			4			5		
	[b] L	M	H	L	M	H	L	M	H	L	M	H	L	M	H	L	M	H
1	28.7	25.0	33.6	66.7	74.5	65.0	3.7	0.5	1.5	0.9	-	-	-	-	-	-	-	-
2	33.3	32.9	36.5	39.5	36.8	32.9	24.8	28.9	29.3	2.3	1.4	1.2	-	-		-	-	
3	52.1	41.1	46.8	27.4	38.6	38.0	6.8	3.8	3.8	13.7	15.8	11.4	-	0.6	-	-	-	-
4	45.8	52.5	52.9	37.5	27.5	29.4	4.2	5.0	2.9	4.2	-	2.9	8.3	15.0	11.8	-	-	-
5	60	50	50	40	50	50	-	-	-	-	-	-	-	-	-	-	-	-

Work

	0		1		2		3		4		5	
	Work	No work	Work	No work	Work	No work	Work	No work	Work	No work	Work	No work
1	30.6	21.1	67.3	77.1	1.8	1.8	0.3	-	-	-	-	-
2	34.8	31.1	36.6	35.1	27.3	30.5	1.4	2.3	-	-	-	-
3	45.0	45.7	36.7	31.4	5.0	2.9	12.9	20.0	0.4	-	-	-
4	55.6	31.3	29.6	37.5	4.9	-	9.9	12.5	-	18.8	-	-
5	-	-	-	-	-	-	-	-	-	-	-	-

Diseases

	0		1		2		3		4		5	
	Healthy	Sick	Healthy	Sick	Healthy	Sick	Healthy	Sick	Healthy	Sick	Healthy	Sick
1	30.3	22.0	67.7	75.8	1.7	2.2	0.3	-	-	-	-	-
2	35.1	29.3	37.0	32.3	26.6	35.4	1.3	3.0	-	-	-	-
3	47.5	35.8	33.6	43.3	4.1	6.0	14.8	13.4	-	1.5	-	-
4	56.2	36.0	26.0	44.0	4.1	4.0	2.7	-	11.0	16.0	-	-
5	-	50.0	-	50.0	-	-	-	-	-	-	-	-

Family status

	0		1		2		3		4		5	
	Single	Relation	Single	Relation	Single	Relation	Single	Relation	Single	Relation	Single	Relation
1	40.0	25.0	57.0	73.5	2.0	1.5	1.0	-	-	-	-	-
2	37.4	33.4	35.1	36.6	26.0	28.6	1.5	1.4	-	-	-	-
3	50.6	43.2	28.9	38.3	6.0	4.0	13.3	14.5	1.2	-	-	-
4	57.1	48.6	28.6	31.4	3.6	4.3	-	2.9	10.7	12.9	-	-
5	57.1	50.0	42.8	50.0	-	-	-	-	-	-	-	-

Quality of life

	0		1		2		3		4		5	
	Low	High	Low	High	Low	High	Low	High	Low	High	Low	High
1	26.4	29.4	71.3	68.7	1.7	1.9	0.6	-	-	-	-	-

	Number of started modules %											
	**0**		**1**		**2**		**3**		**4**		**5**	
2	26.7	39.4	37.9	35.2	32.9	24.5	2.5	0.9	-	-	-	-
3	39.7	49.2	40.5	32.2	3.1	5.6	16.0	13.0	0.8	-	-	-
4	44.9	58.3	28.6	33.3	8.2	-	2.0	2.1	16.3	6.3	-	-
5	42.9	66.7	57.1	33.3	-	-	-	-	-	-	-	-

[a] -age was categorized in 1= young and 2= old based on a mean-split of 44 years

[b] L= low, M= middle, H=high
